# Surgical management of bilateral scapular winging in a previously healthy 10-year-old boy: A case report

**DOI:** 10.1016/j.amsu.2022.104443

**Published:** 2022-08-19

**Authors:** Mohammad G. Ibdah, Salem M. Tos, Narmeen Giacaman, Omar M. Ass'ad, Husam A.T. Saa

**Affiliations:** a Al-Quds University, College of Medicine, Palestine; b Al Makassed Hospital, Jerusalem, Palestine

## Abstract

**Introduction:**

Scapular winging is a pathological condition that occurs due to dysfunction of any scapulothoracic muscles, most commonly the serratus anterior which is innervated by long thoracic nerve.

**Case presentation:**

Herein, we report a 10-year-old boy presented with bilateral scapular winging for a few months, associated with vague discomfort upon shoulder movements. After taking history and performing physical examination and tests as electromyography, he was diagnosed with idiopathic bilateral scapular winging as there was no obvious cause or risk factor for his bilateral scapular winging.

**Discussion:**

Scapular winging causes decrease strength and range of motion of the shoulder, the usual complain of patients is discomfort or the unpleasant cosmetic appearance of scapular prominence, this condition, if left untreated, can cause various negative consequences on the shoulder joint, detailed history and physical exam reinforced by electromyography are crucial in determining the cause of winging, as it has many causes, initial management can be conservative, but various surgical approaches have been described for more severe cases which compromise shoulder function, choice of the surgical approach depends on the underlying cause and overall clinical picture of the patient.

**Conclusion:**

bilateral scapular winging is rare condition, only 26th cases were reported in literature, it occurs due to multiple causes, treatment can be conservative at early stages, but surgical option should be considered when shoulder function and strength become compromised.

## Highlights

• Bilateral scapular winging is quite rare condition that has different causes, only 26 cases were reported in literature.
• Patients usually complain about discomfort, unpleasant cosmetic appearance.
• Management of the choice for this condition depends on the overall clinical picture and patient's condition.
• Split pectoralis major transfer technique has been used in this patient.

## 1 Introduction

Scapular winging or scapula alata is a pathological condition that happens due to impairment in any of the scapulothoracic muscles, mainly the serratus anterior muscle that is supplied by the long thoracic nerve. Scapular winging causes abnormal movements of the scapula over the thorax and leads to altered shoulders’ function as the scapula which supports the upper limb, is not stable anymore [1].

Bilateral scapular winging is commonly caused by systemic disease, as muscular dystrophy, or spinal muscular atrophy [2].

The treatment can be conservative as most cases recover spontaneously. Nerve surgery can be done for acute cases and tendon transfers for chronic cases as nerve procedures are no longer feasible [1]. it is worth noting that surgical options for scapular winging are associated with a higher average cost than conservative management.

Here we report a case of a 10-year-old boy presented with bilateral winging of the scapula most likely due to idiopathic reason.

This work has been reported in line with the SCARE criteria, which is used by authors, journal editors and reviewers to increases the robustness and transparency in reporting surgical cases [18].

## 2 Case presentation

**Fig. 1:**
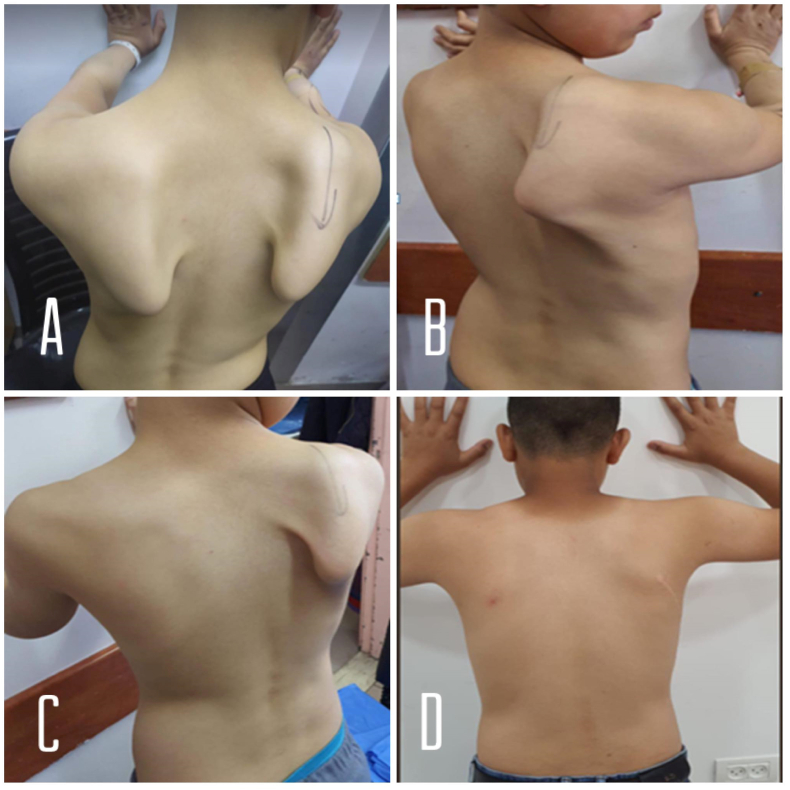
A: Posterior view, bilateral scapular winging are prominent when pushing both hands against the wall with his arms in 90 degrees of anteflexion. **B and C:** near lateral and posterior view, the patient is pushing his right hand against the wall with his arm in nearly 90 degrees of anteflexion. **D:** postoperative picture few months after surgery on follow up, which shows complete resolution of right scapular winging, it also shows complete resolution of the left scapular winging spontaneously.

A 10-year-old boy presented to the clinic complaining of bilateral scapular deformity noted by his family while he was swimming over the past few months. Movement of the affected extremity caused a vague discomfort in the posterior shoulder. The patient was in good overall health and had no personal or family history of hypertension, heart disease, or muscle disease. He denied any known trauma or doing any vigorous routine of weight-lifting within the prior few months. Routine physical examination of the shoulder aimed to assess the static and dynamic scapula posturing and movement, and it revealed a prominent medial border of both scapulas. This finding was accentuated to a blatant deformity when the patient forwardly flexed the right arm (Fig. 1A-D). Neurovascular examination and Neurodiagnostic studies including electromyography and nerve conduction velocity testing of both upper arms were normal. The diagnosis of bilateral winged scapula of idiopathic cause was established and arrangements were made for referral to orthopedic ward to do surgery which is preferred over any other nonsurgical treatment by the patient's parents. The Right scapula winging causing more discomfort and more cosmetic disfigurement than the left, because of that in addition to that the patient is Right-handed dominate, Split Pectoralis Major Transfer for Right scapular winging is planned first.

**Fig. 2:**
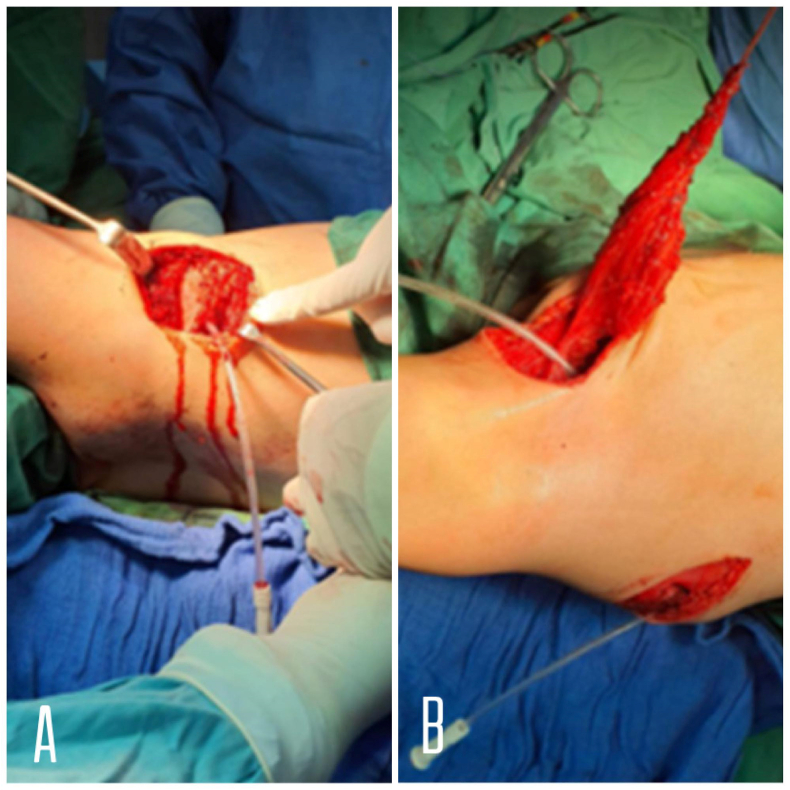
A: Showing exposure of the pectoralis major insertion on the humerus. **B:** Showing the detached insertion part of the pectoralis major tacked with #2 Orthocord that is placed in Krakow fashion in the tendinous and musculotendinous portions of the pectoralis major.

**Fig. 3:**
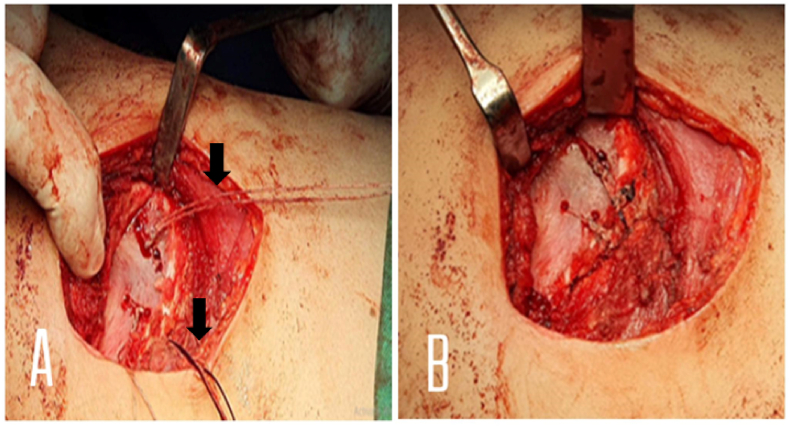
**A:** A series of drill holes are made in the scapula taking care to protect the thoracic cavity below. Sutures are then placed from the dorsal surface through the transferred tendon back up the tendon in a mattress fashion and then out through the matching drill hole. The tendon-gripping sutures are pulled to maximal tension (arrows) before the definitive sutures are placed in the scapula. **B:** The mattress sutures are then sequentially tied along the dorsal surface affecting a secure repair of the transferred sternal pectoralis major tendon.

### 2.1 Intraoperative (Fig. 2, Fig. 3)

With the patient under general anesthesia and in the left lateral decubitus position with the head of the bed elevated 15°, An anterior axillary curvy incision of 6 cm in length was made, electrocautery is used to dissect through the subcutaneous tissue layer. The cephalic vein is mobilized laterally with the deltoid. Wide subcutaneous tissue flaps are created to optimize visualization. Then the surgeon identified the sternal part of right pectoralis major and dissected its tendon at insertion part (lateral lip of bicipital groove). During tendon release, a finger is placed over the underlying biceps long head tendon to prevent iatrogenic injury. After harvest, meticulous hemostasis within the anterior wound is performed.

The arm is repositioned with in-line traction away from the body in approximately 130° of forward elevation to expose the scapula. A second incision was made over the middle portion of the Right inferior scapular angle (5 cm long). And electrocautery is used to dissect through the subcutaneous tissue. By use of digital dissection from the anterior and posterior wounds, a submuscular tissue tunnel is created within the axilla to connect the 2 incisions to properly place the sternal tendon of pectoralis major. At this point, Attention is turned toward assessment of the pectoralis major length-to-transfer distance, and fortunately the length is adequate, so we didn't use autograft tissue harvest, and this help reduce additional morbidity and operative time. After that, using drill, 4 holes were created at the distal part of the scapula. By Lasso suture, the tendon was sutured to the anterior aspect of distal scapula through created holes. A drain applied to the axillary incision. Closure of incisions was done; the operation otherwise was uncomplicated.

### 2.2 Postoperative

The procedure was done with no postoperative complications, stayed at hospital for one day after it, during which he was given painkillers and broad-spectrum antibiotics. The patient was seen several days after the procedure for follow-up, the surgeon removed the sutures and referred him to physiotherapy, he was also seen 3 months and 6 months after the operation, at the last visit, the surgeon noticed spontaneous resolution of the left side winging, the patient and his parents were very satisfied and pleased about the cosmetic result, along with marked improvement of the discomfort and shoulder movements, the surgeon also was fulfilled as the procedure made a significant difference on both functional and aesthetic levels.

## 3 Discussion

Winged scapula results from paralysis of the serratus anterior or trapezius muscles due to damage to the long thoracic or accessory nerves, it causes decreased strength and range of motion of the shoulder [3], patients usually complain about discomfort or the cosmetic appearance of the prominence, however, if left untreated, various consequences can occur including adhesive capsulitis, subacromial impingement, and brachial plexus radiculitis [3]. Most cases of scapular winging are unilateral, which is most commonly caused by damage or impaired innervation to the serratus anterior muscle, innervated by the long thoracic nerve [4]. A winged scapula can also occur as the result of electric shock, cold exposure, blunt trauma, viral infection, vaccination, brachial neuritis, subscapular osteochondroma, and instability of the scapulothoracic joint [3].

Long thoracic nerve is usually exposed and iatrogenically injured in surgeries involving the thoracic region, such as radical mastectomy, resection of the first rib, transthoracic sympathectomy and other procedures in the region [5]. A compressive injury to the nerve may occur from anesthesia or from the phenomenon known as Saturday night syndrome [3].

Physical exam and assessment supported by patient history and background is important to establish the cause, as the winging of scapula is diagnosed simply by visible inspection of the scapula, but the etiology is more challenging [6]. We can classify scapular winging as static or dynamic, this is useful in diagnosis. Static winging is present at rest and is usually due to a fixed deformity like tumors, pseudoarthrosis, exostosis after fracture around shoulder girdle and scoliosis, these are usually cured by operative treatment, with restoration of function most of the time [7].

Dynamic winging occurs with only active or resistive movements and is usually caused by a neuromuscular condition resulting in muscular imbalance. An electromyogram may be needed to confirm nerve damage, while imaging studies may be needed to reveal skeletal or muscular anomalies [7].

Bilateral winged scapula seems to be very rare to the best of our knowledge, as we were able to find only 26 cases reported in the literature, one case after infection with Lyme disease [7], one case as a result of heavy weight lifting [8], one case presented as an association with multiple defects [9], one case of congenital bilateral scapular winging [10], sixteen cases due to facioscapulohumeral muscular dystrophy [[11], [12], [13]], six cases due to unspecified causes [14], so our case is supposed to be the 27th.

Recommended management of winged scapula is initially non-surgical, involving conservative measures like physiotherapy, braces, slings, and other orthotics, especially in patients with minimal symptoms and those who are not fit for surgery, while surgical options are used in more severe cases which compromise shoulder function and strength, or due to patient preference usually because of aesthetic reasons [3].

Several surgical procedures for winged scapula are described, they can be categorized into broad categories of wire fixation, muscle transfer, and nerve grafts [3]. The selection of the appropriate procedure is influenced by the underlying cause and the clinical picture, whether if the deformity is static or dynamic.

In our case, a muscle transfer technique was chosen, specifically split pectoralis major transfer which is described above. This technique of treatment has proven to be effective in treating scapular winging. There are multiple techniques available for this procedure, with little evidence to support one over the other [15]. Most patients undergoing this procedure have regained full range of motion upon recovery [16], most frequent reported complications for this operation is transfer failure, infection, unsatisfactory cosmetic result, glenohumeral stiffness [17].

## 4 Conclusion

Scapular winging is a dysfunction involving the stabilizing muscles of the scapula, most commonly the serratus anterior or trapezius, resulting in abnormal motion of the scapula and protrusion of its medial or lateral borders, diagnosis is made clinically by physical exam and assessment, winging is usually unilateral but can be bilateral in rare occasions, especially in muscular dystrophies. Patients usually complain about discomfort or the unpleasant appearance, management can be conservative and non-surgical initially as most cases seems to recover spontaneously, if not, it is recommended to treat the condition operatively because several consequences can ensue if left untreated, including adhesive capsulitis, subacromial impingement, and brachial plexus radiculitis. Various surgical techniques have been described, the choice depends on the underlying cause and overall clinical picture of the patient.

## Ethical approval

Informed consent was signed from the patient's parents for publication.

## Sources of funding

No funding or grant support.

## Authors’ contributions

Study concept or design: Husam A.T. Saa. Writing the manuscript: Mohammad G. Ibdah, Salem M. Tos, Narmeen Giacaman, Omar M. Ass'ad. Review & editing the manuscript: Mohammad G. Ibdah, Salem M. Tos.

## Consent

Written informed consent was obtained from the patient's parents for publication of this case report and accompanying images. A copy of the written consent is available for review by the Editor-in-Chief of this journal on request.

## Guarantor

Mohammad G. Ibdah.

## Provenance and peer review

Not commissioned, externally peer reviewed.

## Declaration of competing interest

The authors declare no conflicts of interest.
